# The Contribution of Physiological and Accelerated Aging to Cancer Progression Through Senescence-Induced Inflammation

**DOI:** 10.3389/fonc.2021.747822

**Published:** 2021-09-21

**Authors:** Jorge Morales-Valencia, Gregory David

**Affiliations:** ^1^Department of Biochemistry and Molecular Pharmacology, New York University School of Medicine, New York, NY, United States; ^2^NYU Cancer Institute, New York University School of Medicine, New York, NY, United States; ^3^Department of Urology, New York University School of Medicine, New York, NY, United States

**Keywords:** cancer, aging, senescence, inflammation, SASP

## Abstract

Senescent cells are found to accumulate in aged individuals, as well as in cancer patients that receive chemotherapeutic treatment. Although originally believed to halt cancer progression due to their characteristic growth arrest, senescent cells remain metabolically active and secrete a combination of inflammatory agents, growth factors and proteases, collectively known as the senescence-associated secretory phenotype (SASP). In this review, we discuss the contribution of senescent cells to cancer progression through their ability to alter cancer cells’ properties and to generate a microenvironment that promotes tumor growth. Furthermore, recent evidence suggests that senescent cells are able resume proliferation and drive cancer relapse, pointing to the use of senolytics and SASP modulators as a potential approach to prevent tumor resurgence following treatment cessation. Thus, a better understanding of the hallmarks of senescence and the impact of the SASP will allow the development of improved targeted therapeutic strategies to leverage vulnerabilities associated with this cellular state.

## Introduction

Age is by far the most significant risk factor for cancer development, as the incidence of most cancers exponentially increases with age (1). Accordingly, as of 2017, cancer remained the leading cause of death in both males and females aged 60-79 years (2). The exponential increase in tumor incidence with age suggests that some age-associated factors are yet to be identified as contributors to cancer. Hallmarks of aging shared in the context of cancer include epigenetic changes, mitochondrial dysfunction, changes in proteostasis, among others (3). Such observations raise the idea that hallmarks of aging are functionally relevant to the properties of cancer cells (4).

One of the hallmarks of aging that could provide a connection with cancer is the development of the inflammaging. Inflammaging refers to the accumulation of inflammatory markers in the blood of older individuals in the absence of microbial infection (5, 6). It is estimated that approximately 20% of tumors result at least in part from prolonged and persistent inflammation (7). This response to chronic inflammation not only contributes to the pathogenesis of cancer but also to cardiovascular diseases, metabolic syndromes, neurodegeneration, diabetes and certain infections (8, 9). What triggers the seemingly sterile inflammation that develops with age remains largely unclear. Several cellular and molecular processes contribute to the development of inflammaging, including the accumulation of damaged cellular components (10), harmful products produced by oral or gut microbiota (11), defective autophagy (12) and finally, cellular senescence (9).

Cellular senescence is a stable form of cell exit which overlaps with quiescence or terminal differentiation at the molecular level. Senescence represents a response to different sources of stress that converge towards the activation of the DNA Damage Response (DDR) and ultimately, growth arrest. Sources of stress known to trigger senescence include telomere shortening, oncogene expression and exposure to chemotherapeutic agents or irradiation (13–15). For a general view of the molecular and cellular underpinnings of cellular senescence, we refer the reader to these comprehensive reviews (16–19). Despite a seemingly irreversible growth arrest, senescent cells remain metabolically active and secrete a discrete set of inflammatory agents, growth factors, and proteases, collectively known as the Senescence-Associated Secretory Phenotype (SASP) (20–22). While the composition of the SASP remains largely comparable in different biological contexts, recent studies have uncovered some heterogeneity depending in part on tissue of origin, and the nature of the senescence-inducing stimulus. Indeed, transcriptomic analysis of six fibroblast cell lines induced to senesce by three different stimuli (replicative, ionizing radiation and oncogene overexpression) revealed that SASP factors exhibited significant heterogeneity (23). For example, oncogenic Ras-overexpressing senescent cells secrete a higher level of IL-6 and IL-1β cytokines compared to X-irradiated or replicative senescent cells (24). Moreover, the SASP was significantly amplified by oncogenic Ras or loss of p53 function in epithelial tumor cells exposed to DNA-damaging chemotherapy (24). Additionally, the composition of the SASP varies over time following exposure to oncogenic stimuli, with a switch between two different types of secretome, including an early NOTCH-driven SASP followed by the canonical CEBPα-driven SASP (25). Finally, late senescence is characterized by the activation of a retrotransposon-induced type I IFN response, thus modifying the cytokine profile comprising the SASP at this stage (26). Overall, these studies reveal that the SASP is not a static phenotype but can develop into a range of secretomes with distinct quantitative and qualitative features.

The impact of the SASP on tumor progression remains ill-defined, mainly due to the challenge of uncoupling SASP production from other senescence associated phenotypes, such as cell cycle exit. The SASP has been suggested to bear antitumorigenic effects including reinforcement of senescence in an autocrine and paracrine manner (22, 27). However, by modifying the tissue microenvironment and altering the function of surrounding cells, the SASP is also thought to drive age-associated pathologies and favor tumorigenesis (13, 28). Here, we focus on the contribution of senescence, a hallmark of aging, and its associated inflammation on cancer progression. In particular, we highlight how different senescence triggers and SASP components induce some of the most potent drivers of aggressiveness in cancer such as epithelial-to-mesenchymal transition (EMT), increased migration and emergence of cancer stem cells (CSCs). Lastly, we discuss potential therapeutic opportunities to reduce such adverse effects.

## Age-Dependent Accumulation of Senescent Cells as a Driver of Cancer Progression

Senescence is a cellular response that limits the proliferation of aged or damaged cells (13, 29). Indeed, various markers of senescent cells accumulate in aged mammals (30–32). As cells proliferate, telomere erosion ultimately results in the exposure of uncapped chromosome ends that resemble a double strand break, leading to the recruitment of ataxia telangiectasia mutated (ATM). ATM activation, in turn, promotes the stabilization of p53 and subsequent transcriptional activation of p21, preventing entry into S-phase (33). Other triggers of age-dependent senescence include: epigenetic dysregulation, DNA damage and mitochondrial dysfunction (29). At the organismal level, the aging process is characterized by an exponential accumulation of senescent cells that stems from the imbalance between their production and their clearance (20). The accumulation of senescent cells during the aging process raised the possibility that senescence directly contributes to age-related pathologies. Thus, manipulating the clearance of senescent cells to levels that offset their production in aged individuals could be beneficial for health. Accordingly, clearance of senescent cells, through the elimination of p16(Ink4a)-expressing cells, decreased the incidence of age-associated phenotypes in a mouse model of accelerated aging (34). Importantly, similar outcomes were obtained in experiments using naturally aged mice (35).

Our understanding of how senescent cells promote age-associated phenotypes remains incomplete. As mentioned above, the accumulation of senescent cells during natural aging is accompanied by the systemic secretion of SASP factors in the organism. For example, the concentration of the canonical SASP cytokine IL-6 is low or undetectable in healthy young and middle-aged adults. By contrast, elevated levels have been reported in older adults (36). As a likely result of SASP production, sterile inflammation can be detected in aged mammals, with various effects on numerous diseases, and in particular on tumor initiation and progression. Inflammation can be functionally linked to tumorigenesis either by generating a Tumor Microenvironment (TME) that favors cancer cell growth, or by directly affecting cancer cells’ intrinsic properties. A senescence-dependent immunosuppressive TME that promotes tumor growth and chemoresistance was observed in a prostate cancer model (37). In the PTEN-deleted mouse model, senescent tumors were characterized by an infiltration of myeloid derived suppressor cells (MDSCs) in absence of natural killer (NK) cells (37). Moreover, the contribution of the SASP factors IL-6 and IL-8 in the modulation of the tumor microenvironment (TME) is well established. For example, IL-6 secretion by senescent skin stroma has been shown to fuel cancer progression by promoting the recruitment and expansion of immunosuppressive myeloid cells within the TME (38). Lastly, IL-8 secreted by senescent cancer-associated fibroblasts (CAFs) is able to promote invasion and metastasis in mouse models of pancreatic cancer (39).

In addition to promoting a tumor permissive environment, the SASP can have a direct effect on cancer cells and enhance their degrees of aggressiveness. Incubation with conditioned medium from senescent fibroblasts stimulates hyperproliferation and progression of premalignant and malignant epithelial cells (40). Similarly, the SASP secreted by senescent human umbilical cord mesenchymal cells enhanced the proliferation of breast cancer cells (41). Moreover, exposure to secreted factors from aged fibroblasts drove therapeutic resistance of melanoma cells by promoting local invasion and metastasis (42).

Administration of individual SASP factors is often sufficient to promote such effects in cancer cells, pointing to the possibility that exposure to the SASP may further enhance cancer aggressive traits as a result of the additive effect of each SASP component. Accordingly, exposure of breast cancer cells to recombinant IL-6 or IL-8 enhances their migration and invasion properties, as well as induces mammosphere formation, a readout for CSC potential (43). Bavik et al. showed that aging-related secretion of Fibroblast Growth Factor 7 (FGF7) and Hepatocyte Growth Factor (HGF) by senescent prostate fibroblasts, stimulated epithelial cell proliferation, contributing to prostate neoplasia progression (44). Finally, CXCR2, a chemokine receptor for SASP factors, whose expression is increased during replicative senescence (45), is a poor prognostic marker in lung adenocarcinoma and its expression is associated with tumor invasion and metastasis (46).

Overall, age-related accumulation of senescent cells and the impact of the SASP on cancer cells and inflammatory signaling may result in a tumor microenvironment increasingly conducive of tumor development and growth. This could at least partially account for the rise in cancer incidence and deaths in aged patients, outweighing some of the observed antitumorigenic features of the SASP.

## Age-Independent Contribution of the SASP To Cancer Progression

In addition to normal chronological aging, some genetic disorders result in premature or accelerated aging. In these syndromes, patients present common age-associated phenotypes, including heart failure, atherosclerosis and decreased lifespan. Werner’s Syndrome (WS) is one of several rare premature aging syndromes with links to cancer, and its etiology stems from the loss of helicase and exonuclease activities of the WRN protein (47). Patients affected with this syndrome exhibit age-associated pathologies early in life including the development of an unusual spectrum of tumors such as soft tissue sarcomas, thyroid cancers and meningiomas (48). Importantly, cells derived from WS patients undergo premature replicative senescence when cultured *ex vivo* (49). Accordingly, cells rendered senescent by WRN knock-down also exhibit a SASP phenotype, capable of triggering an increased proliferation and enhanced protumorigenic properties in both HFF-1 and MCF7 cells (50). Of note, senescence is also detected in skin, muscle and cardiovascular cells of patients with Hutchinson-Gilford Progeria Syndrome (HGPS) (51).

Apart from accelerated aging related processes triggered by genetic mutations, exogenous sources of stress can also induce a senescence response. For example, exposure to chemotherapeutic drugs results in the accumulation of senescent cells in the process now known as Therapy-Induced Senescence (TIS). Because senescence is largely characterized by a stable growth arrest, TIS was originally proposed as a strategy to halt cancer progression by inducing senescence in cancer cells to limit their uncontrolled proliferation (52). Schmidtt and colleagues provided evidence that senescence could be induced upon cyclophosphamide treatment in a mouse model of lymphoma (53). Indeed, senescent cancer cells have been detected in various mouse models and cell lines in response to chemotherapy (54, 55). Likewise, patients treated for breast cancer by chemotherapy exhibit increased expression of senescence markers long after treatment cessation (56).

However, accumulating evidence indicates that senescent cancer cells are capable of reentering the cell cycle and promoting tumor relapse and metastasis, possibly by engaging a pro-inflammation program. *In vitro* studies demonstrated that p53-null lung cancer cells induced to senesce upon administration of chemotherapeutic drugs were able to spontaneously resume proliferation (57), raising the possibility that senescent tumor cells may promote the emergence of proliferative clones and tumor relapse upon treatment termination. Additionally, Duy and collaborators showed that exposure to chemotherapy could rather induce a senescent-like phenotype, which differs from classic senescence by the temporary growth arrest it promotes. Intriguingly, these cells express a SASP comparable to the one defined in “fully-senescent” cells (see below). The inflammatory response exhibited by these cells may protect them from the cytotoxic effects of Ara-C, providing a cellular basis for acute myeloid leukemia (AML) relapse after chemotherapy (58). Such escape from senescence is not unprecedented: Yang et al. demonstrated that spontaneous reversal of doxorubicin-induced senescence into a proliferative state enhanced invasive and migratory properties, allowing revertants to out-compete parental cells in *in vivo* tumor growth experiments (55). Likewise, H460 non-small cell lung, HCT116 colon and 4T1 breast tumor cells lines induced to senesce by exposure to either etoposide or doxorubicin were able to resume proliferation and form tumors when implanted in either immunodeficient and immunocompetent mice (59).

In a model of B-cell lymphoma, cells released from doxorubicin-induced senescence showed highly aggressive growth potential, compared to cells that had been exposed to chemotherapy but were never senescent (60). Furthermore, this study reveals that senescent tumors comprise an increased proportion of CSCs, known to contribute to cancer relapse and metastasis (61). Similarly, doxorubicin-induced senescence in hepatocellular carcinoma cells was accompanied by a significant increase in expression of reprogramming genes SOX2, KLF4, c-MYC as well as other liver stemness-related genes. These features correlated with an increased *in vivo* tumor-forming ability (62). In these examples, the specific impact of SASP secretion on the emergence of aggressive cancer phenotypes has not been investigated. It is tempting to speculate that the inflammatory milieu generated upon exposure to the SASP may contribute, in part, to the relapse observed following the cessation of chemotherapy treatment.

Several studies have demonstrated that cancer cells induced to senesce upon exposure to chemotherapy express a robust SASP, which confers aggressive tumorigenic properties when incubated with cancer cells. Coppé et al. showed that both XRA and mitoxantrone-induced SASP production in different prostate epithelial cancer cell lines. Additionally, the SASP factors IL-6 and IL-8 secreted by XRA-induced senescent fibroblasts, promoted an epithelial-to-mesenchymal transition (EMT) and enhanced invasiveness of premalignant epithelial cells (24). Likewise, the SASP produced by chemotherapy-induced senescent mesothelioma cells can trigger the emergence of EMT and chemoresistant cell subpopulations (63). Moreover, the SASP produced by tumor cells induced into senescence by cisplatin or doxorubicin treatment is capable of increasing proliferation of tumor cells (64, 65). For instance, MMPs secreted from bleomycin-induced senescent human fibroblasts increase the proliferative capacity of MDA-MB-231 breast cancer cells in nude mice xenograft transplants (66). Finally, in multiple myeloma, exposure to the SASP produced by cisplatin-induced-senescent cells results in an emergence of CSCs (67).

Additionally, other health-related factors promote the accumulation of senescent cells in the organism. The accumulation of senescent cells in adipose tissue correlates with increased levels of obesity, particularly in visceral fat (68). Morbidly obese subjects can have a 30-fold increase in the burden of senescent preadipocytes compared to non-obese subjects (68). In this context, the presence of senescent, pro-inflammatory cells could have profound clinical consequences due to the large size of the adipose tissue. Noticeably, the rates of obesity are rising rapidly in younger patients, which may contribute to the increasing rates of cancer in this population (69). Accumulating evidence links obesity-associated inflammation and cancer incidence and progression. Similar to aged individuals, obese patients express elevated levels of the SASP components IL-6, IL-8 and TNF-α (70, 71). IL-6 from adipose stromal cells can promote breast cancer cell proliferation and migration (72). Moreover, a role for senescence and particularly the SASP, has been elucidated in obesity-associated hepatocellular carcinoma (HCC). Obese mice challenged with deoxycholic acid (DCA) exhibit increased levels of senescent cells and inflammatory markers in the liver, whereas mice lacking a SASP inducer or depleted of senescent cells present lower HCC occurrence (73). Apart from cancer, obese individuals present an increased risk of developing other diseases associated with aging such as type 2 diabetes, cardiovascular disease, osteoarthritis among others (74, 75). The contribution of the SASP in the inception of disease in these contexts remains largely unexplored.

**Figure 1:**
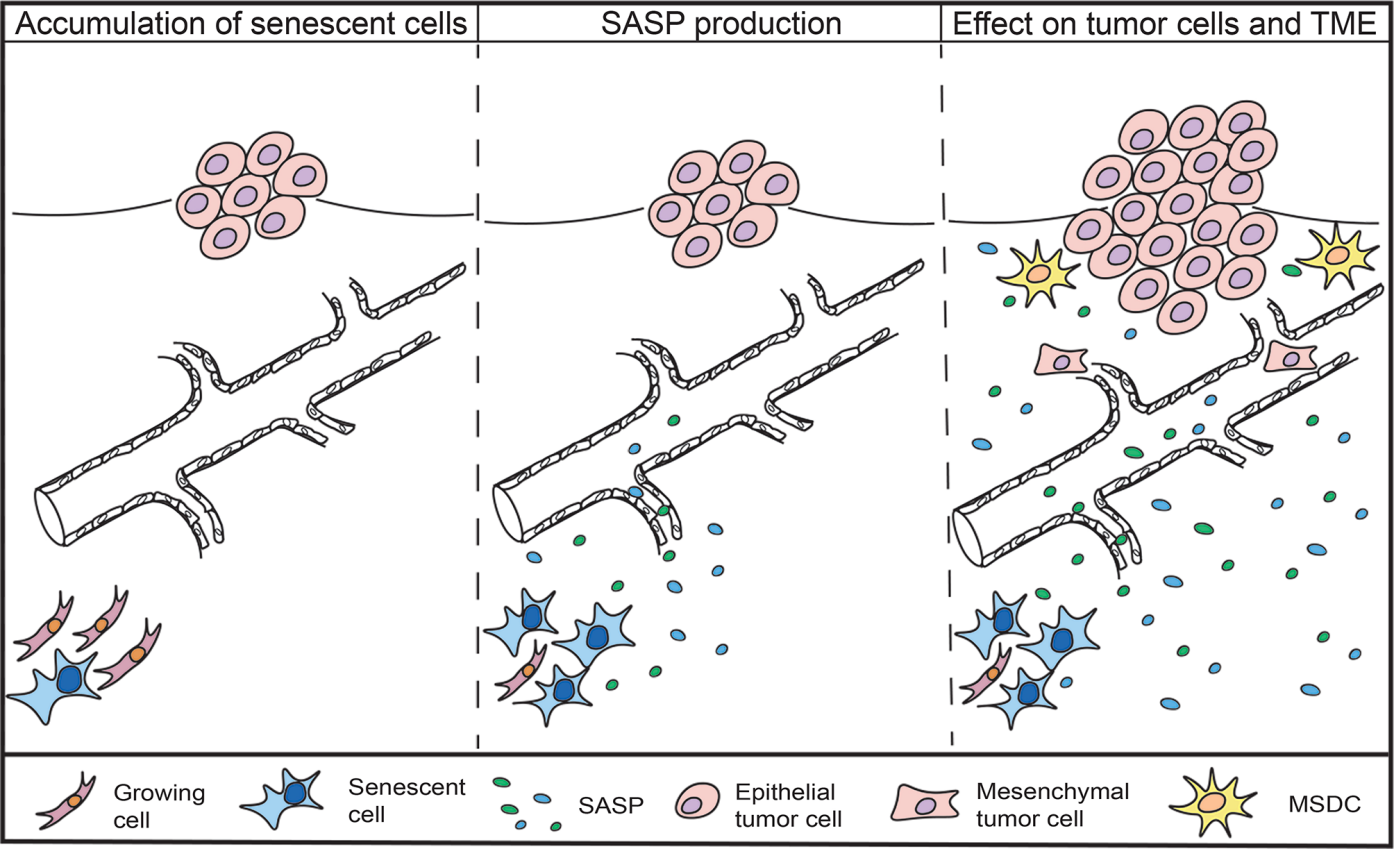
Accumulation of senescent cells and the impact of the SASP on cancer cells and TME. Senescent cells are found to accumulate in aged and obese individuals, as well as in cancer patients that receive chemotherapeutic treatment. Senescent cells remain metabolically active and secrete a combination of inflammatory agents, growth factors and proteases, collectively known as the senescence-associated secretory phenotype (SASP). The SASP can promote the proliferation of tumor cells, induce epithelial-to-mesenchymal-transition, recruit immunosuppressive cells and increase the emergence of cancer stem cells.

Overall, evidence suggests that the accumulation of senescent cells in aged individuals and the accompanying SASP production could be linked to the increased tumor incidence and cancer relapse also observed in patients suffering from accelerated aging syndromes and severe obesity (**Figure 1**).

## Therapeutic Opportunities

In light of the seemingly opposing effects of senescence-associated phenotypes on tumorigenesis, uncoupling the detrimental aspects of senescence from its beneficial properties could provide novel therapeutic strategies for cancer treatment (22). In the absence of an established strategy to specifically blunt the SASP, the selective elimination of senescent cells by senolytics or other non-discriminatory approaches may provide opportunities to mitigate age-associated cancer progression and other phenotypes (76). *In vivo* evidence also supports this idea, the Van Deursen group showed that clearance of senescent cells decreased the incidence of aging-associated disorders in mice (34). Additionally, using a transgenic mouse model that allows for the tracking and elimination of senescent cells, the Campisi group has shown that elimination of doxorubicin-treated senescent fibroblasts can prevent or delay cancer relapse and metastasis and reduce side effects of the drug such as cardiac dysfunction and BM suppression (77). Pharmacological elimination of senescent cells proved to have similar results: Administration of Navitoclax (ABT-263), a BCL-2 inhibitor that causes senescence cells to undergo apoptosis, significantly increases the efficacy of some chemotherapeutic regimens in models of lymphoid malignancies including B-cell lymphoma and multiple myeloma (78). In a phase I clinical trial for Chronic Lymphocytic Leukemia (CLL), 35% of patients with relapsed disease treated with Navitoclax achieved a partial response (79). Additionally, ABT-263 administration was proven to be beneficial against other age-related diseases such as neurodegenerative disease, osteoarthritis and heart failure, and the progression of some solid tumors (80–82). Similarly, Dasatinib, a tyrosine kinase inhibitor, can function as a senolytic when combined with Quercetin, reducing senescent cell abundance *in vivo* (83). D+Q treatment delayed death from cancer in mice and decreased complications of diabetes and obesity and other age-related pathologies like osteoporosis (84, 85). Unsurprisingly, these effects of senolytics correlated with a decrease in SASP production. For example, the levels of SASP factors, IL-6, IL-8, MCP-1 and GM-CSF, decreased significantly in conditioned media from cultures treated with D+Q (86).

Another prospective strategy is the selective ablation of the SASP, which would circumvent the possible organ collapse resulting from the simultaneous elimination of a large number of senescent cells in aged or disease organs. Among the pathways that control SASP production, IL-1α corresponds to an essential upstream regulator of the inflammatory SASP (87, 88). Therefore, drugs that target the IL-1 pathway, such as the IL-1 receptor inhibitor Anakinra, may prove to be effective therapeutics in a context-dependent manner. Anankira has been suggested for treatment of active myeloma (89). IL-1R neutralization during early stages of tumorigenesis has been shown to reduce the incidence of methylcholanthrene (MCA)-induced fibrosarcoma in mice (90). Accordingly, IL-1α deletion delayed cancer progression in a mouse model of pancreatic cancer; in this model, a decrease in the number and grade of PanIN (Pancreatic Intraepithelial Neoplasia) lesions was observed in IL-1α-null mice compared to their WT counterparts (88). Similar to IL-1α inactivation, mTOR inhibition or rapamycin administration diminished the SASP without reversing senescent-associated cell cycle exit. The inhibition of mTOR suppressed the SASP by downregulating MAPKAPK2 translation in cells undergoing oncogenic-induced senescence (OIS), replicative senescence and γ-irradiation-induced senescence. Moreover, rapamycin, was shown to reduce IL-1R-dependent SASP by inhibiting the translation of IL-1α mRNA, which reduced transcription of inflammatory genes regulated by NF-kB (91, 92).

Additional signaling pathways, transcription factors and regulators have been shown to control the SASP. The pharmacological modulation of such pathways could thus represent novel therapeutic avenues to explore. For example, the autophagy-lysosome pathway has been implicated in establishing the SASP through GATA4-regulation, which in turn activates NFkB and thus, the SASP (93). Furthermore, senescent cells are dependent on fatty acid synthase (FASN) upregulation for their *de novo* lipid synthesis and maintenance of their high metabolic activity, which has imnplications for SASP production. Indeed, treatment of senescent cells with C75, a FASN inhibitor, reduced secretion of SASP factors IL-1α, IL-1β and IL-6 by senescent hepatic stellate cells (HSCs) (94). Likewise, treatment of senescent oral keratinosites with Y-27632 resulted in inactivation of Rho kinase (ROCK), a downstream target of the small GTP-binding protein Rho, decreasing the abundance of secreted IL-1α, IL-1β, IL-6 and IL-8 without compromising the cell growth arrest (95). Other therapeutic opportunities include MLL1 inhibition, which disables the SASP by preventing the activation of genes involved in the DDR (96) and BRD4 inhibition, which prevents the activation of super-enhancers controling SASP genes’ transcription (97). However, lack of specificity may complicate clinical use for some SASP regulators, and unwanted effects resulting from the inhibition of specific pathways need to be further investigated in the context of SASP production and cancer promotion. For instance, nicotinamide mononucleotide (NMN), a supplement used to alleviate certain age-related conditions, paradoxically enhances the proinflammatory SASP and promotes pancreatic ductal adenocarcinoma (PDAC) progression in a mouse model by increasing the NAD^+^/NADH ratio (98). For a more detailed review on SASP modulation we suggest the following review (99).

In biological contexts where senescence or the SASP have beneficial effects, delineating the downstream effectors of the SASP that contribute to cancer progression may also be therapeutically advantageous. In these cases, one could develop therapeutics that specifically inhibit the protumorigenic portion of the SASP. For example, targeting CCL2, secreted by stromal senescent cells, was effective in suppressing progression of colorectal cancer progression (100). Likewise, amphiregulin (AREG) targeting, produced by senescent cells in response to anticancer treatments, diminished cancer resistance and averted the creation of an immunosuppressive TME (101). Overall, a better understanding of the complexity of the SASP and its components as they relate to cancer progression is warranted to develop informed therapeutic interventions.

## Conclusion

**Figure 2:**
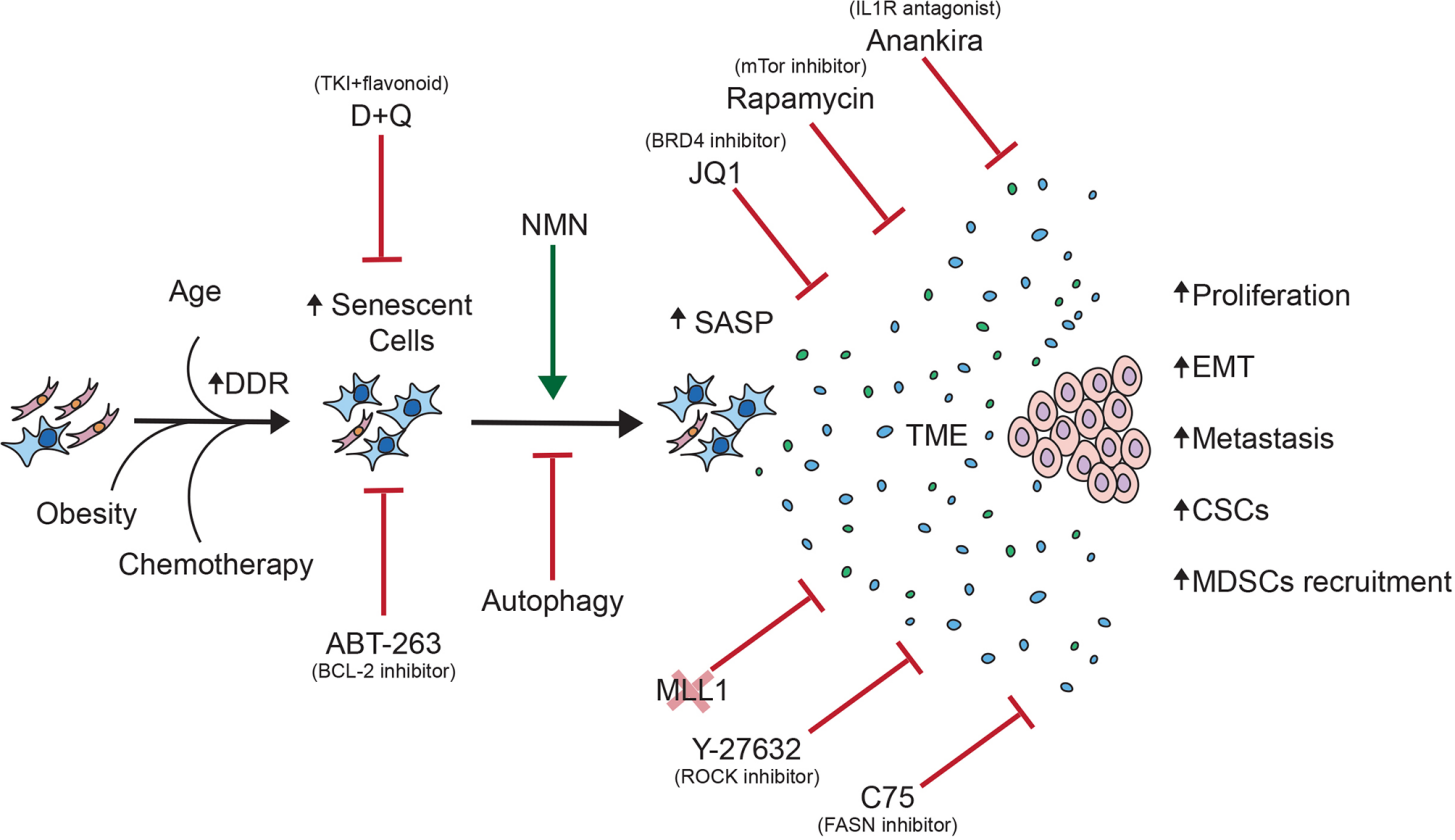
Overview of the contribution of senescent cells to cancer progression. This schematic summarizes the pro-tumorigenic effects of the SASP on tumor cells as well as therapeutic opportunities to abrogate said effects.

In this review, we discussed the contribution of the pro-inflammatory SASP produced by senescent cells to cancer progression, as well as therapeutic opportunities that could abrogate the deleterious effects of the SASP on tumor cells (**Figure 2**).

Senescent cells accumulate with aging and represent a source of the inflammaging, a low-grade chronic inflammation that contributes to age-related pathologies, also observed in patients with progeria and related syndromes, cancer patients receiving cytotoxic therapies and obese patients. Senescent cells and their pro-inflammatory SASP are likely to contribute to the increase mortality observed in the elderly and chronically-diseased upon a cancer diagnosis. Whether the SASP sensitizes the aging population to other diseases remains to be fully explored. Of note, senescent cells have been shown to respond to exposure to SARS-CoV-2 antigens by secreting higher levels of inflammatory factors than the corresponding proliferating cells (102). The contribution of senescence to the increased vulnerability to SARS-CoV-2-mediated mortality observed in the elderly and patients with co-morbidities has yet to be elucidated.

Originally thought of as a mechanism to prevent tumor cell proliferation, senescence induction can also exhibit pro-tumorigenic properties by directly affecting tumor cells behavior and generating an immunosuppressive TME. Additionally, recent evidence suggests that senescent cells can drive cancer relapse upon cancer treatment suspension. Therapeutic strategies that couple senescence induction with SASP abrogation may be proven beneficial. However, because of the SASP heterogeneity, designing novel therapeutic strategies for the treatment of cancer will undoubtedly depend on cell and tissue context, warranting more studies. Targeting downstream SASP effectors may represent a more efficient and specific alternative. In conclusion, we believe that studies that improve the understanding of cellular senescence and SASP composition will enhance current cancer therapies and will make it possible for targeted therapeutic opportunities to be developed, benefiting patients affected with cancer.

## Author Contributions

JM-V wrote the review under the guidance of GD. All authors contributed to the article and approved the submitted version.

## Funding

This work was funded by the NIH/NCI (R21CA246416) and the NYSTEM Institutional Training Grant (C322560GG).

## Conflict of Interest

The authors declare that the research was conducted in the absence of any commercial or financial relationships that could be construed as a potential conflict of interest.

## Publisher’s Note

All claims expressed in this article are solely those of the authors and do not necessarily represent those of their affiliated organizations, or those of the publisher, the editors and the reviewers. Any product that may be evaluated in this article, or claim that may be made by its manufacturer, is not guaranteed or endorsed by the publisher.
